# Prognostic and clinicopathologic significance of circZFR in multiple human cancers

**DOI:** 10.1186/s12957-022-02733-9

**Published:** 2022-08-26

**Authors:** Zhongyue Liu, Wenchao Zhang, Chao Tu, Wenyi Li, Lin Qi, Zhiming Zhang, Lu Wan, Zhimin Yang, Xiaolei Ren, Zhihong Li

**Affiliations:** 1 Department of Orthopedics, The Second Xiangya Hospital, Central South University, No.139 Middle Renmin Road, Changsha, Hunan 410011 People’s Republic of China; 2 Hunan Key Laboratory of Tumor Models and Individualized Medicine, The Second Xiangya Hospital, Central South University, No.139 Middle Renmin Road, Changsha, Hunan 410011 People’s Republic of China

**Keywords:** CircZFR, Cancer, Prognosis, Clinicopathology, Meta-analysis

## Abstract

**Background:**

Abnormally expressed in diverse cancers, circZFR has been correlated with clinical outcomes of cancer patients. Aim of this meta-analysis was to elucidate the prognostic role of circZFR in multiple human malignancies.

**Methods:**

Literature retrieval was conducted by systematically searching on Pubmed, Web of Science, and the Cochrane Library up to December 2nd, 2021. Hazard ratios (HRs) or odds ratios (ORs) with 95% confidence intervals (CIs) were pooled to evaluate the association between circZFR expression and overall survival (OS). The reliability of the pooled results was assessed through sensitivity analysis and the publication bias was measured by Begg’s and Egger’s test.

**Results:**

A total of seventeen studies comprising 1098 Chinese patients were enrolled in this meta-analysis. Results demonstrated that high circZFR expression was correlated with an unfavorable OS (HR = 2.14, 95% CI 1.74, 2.64). High circZFR expression predicted larger tumor size (OR = 2.79, 95% CI 1.52, 5.12), advanced clinical stage (OR = 3.38, 95% CI 1.49, 7.65), tendentiousness of lymph node metastasis (LNM) (OR = 3.08, 95% CI 2.01, 4.71), and malignant grade (OR = 3.18, 95% CI 1.09, 9.30), but not related to age, gender, and distant metastasis (DM).

**Conclusions:**

High circZFR expression was associated with unfavorable OS and clinicopathologic parameters including tumor size, clinical stage, LNM, and histology grade, implicating a promising prognostic factor in cancers.

**Supplementary Information:**

The online version contains supplementary material available at 10.1186/s12957-022-02733-9.

## Background

In the human genome, about 93% of DNA sequences can be transcribed into RNA, of which only less than 2% are translatable, and the rest are specified as non-coding RNA (ncRNA) [1]. Circular RNAs (circRNAs), as a member of ncRNAs, were first discovered in RNA viruses and then in eukaryotes in the 1970s [2, 3]. CircRNAs are characterized by highly conserved and the unique covalently closed loop structure without free 5′ cap and 3′ tail, which avoids degradation by exonuclease and differentiates it from other ncRNAs such as lncRNA and miRNA. The expression of circRNAs has tissue, time and disease specificity. Most circRNAs are derived from exons and located in the cytoplasm, while a few are directly cycled by introns and located in the nucleus. However, the mechanism of circRNAs formation remains unclear [4]. Currently, many studies have shown that circRNAs exert their biological functions mainly by acting as miRNA molecular sponges through competing endogenous RNA (ceRNAs), interacting with proteins and regulating gene splicing or transcription, and translating into proteins or peptides that perform epigenetic functions. CircRNAs are involved in many physiological and pathological processes, such as aging [5], diabetes [6], and various cancer [7–9]. It is worth noting that the circRNAs play diverse roles in tumorigenesis and development, which can regulate tumor proliferation [10], metastasis [11], and drug resistance [12, 13].

Circular RNA zinc finger RNA-binding protein (CircZFR), a transcription product of zinc finger RNA-binding protein (ZFR) gene, is mapped to chromosome 5p13.3, and has been identified as a novel oncogenic or suppressing modulator in several human cancers [14]. Recently, circZFR has been reported to be associated with the disease progression in hepatocellular carcinoma (HCC) [15], lung cancer (LC) [16], papillary thyroid cancer (PTC) [17], bladder cancer (BlC) [18], breast cancer (BrC) [19, 20], gastric cancer (GC) [21], colorectal cancer (CRC) [22], and esophageal squamous cell cancer (ESCC) [23]. Most studies reported that circZFR acted as oncogenic function to promote the cancer progression. However, the small clinical sample size limited the clinical significance of circZFR. Here, we made the meta-analysis of circZFR in various cancers to emphasize its clinical application potential.

## Methods

### Publication search strategy

This meta-analysis was projected, reviewed, and reported based on the Preferred Reporting Items for Systematic Reviews and Meta-Analysis (PRISMA) checklist. A comprehensive research was conducted by two independent authors (ZY L and WC Z) in PubMed, Web of Science, and the Cochrane Library up to December 2nd, 2021. The Search strategy were listed as follows: “circZFR” OR “circ_ZFR” OR “circ-ZFR” OR “circRNA ZFR” OR “circular RNA ZFR” OR “circ_0072088” OR “circ_0072083” OR “Circ_103809” OR “circRNA_103809” OR “Hsa_circRNA_103809” OR “Circular RNA hsa_circRNA_103809” (the detailed search strategy for each of the databases was presented in the supplementary materials). The additional research of citation lists of included publications was manually identified for relevant articles.

### Inclusion and exclusion criteria

The inclusion and exclusion criteria are presented as follows. Inclusion criteria: (1) patients definitely diagnosed with cancer by histopathology; (2) studies focusing on the clinical prognostic or clinical value of circZFR in any type of cancers; (3) circZFR were assigned to high expression group (high) or low expresssion group (low) according to its relative expression level; (4) studies providing sufficient information about the correlation between circZFR expression level and overall survival (HRs with 95% CIs) or clinical characteristics (age, gender, stage, grade, and so on). Exclusion criteria: (1) duplicate publications; (2) studies focusing on the structures or functions of circZFR and without clinical prognostic information; (3) available data are not extractable; (4) studies without original data like reviews and meta-analysis.

### Data extraction and quality assessment

All the enrolled studies were independently evaluated by two investigators (ZY L and WC Z) and discrepancies were settled by consultation with a third investigator (XL R). All the included studies are non-RCT. The baseline data extracted from the included studies were as follows: (1) first author, study year, country, cancer type, clinical stage, tumor size, cut-off value, follow-up time, detection method, adjuvant therapy before surgery, survival analysis method, and outcome measure method; (2) hazard ratios (HRs) or odds ratios (ORs) with 95% confidence intervals (CIs) of circZFR for OS or clinicopathologic parameters. We used the software Engauge Digitizer (version 4.1) to calculate the HRs with 95% CIs according to Kaplan-Meier curves when they were not presented in the studies directly. The quality of the eligible literature was evaluated by NOS, whose score up to 7 indicated high quality of the study.

### Data synthesis and statistical analysis

The statistical analyses were conducted by STATA software (version 12.0) and Review Manager (RevMan 5.3). HRs or ORs with corresponding 95% CIs were used to describe the relationship between circZFR expression and the prognosis or clinical characteristics. The chi-squared test and *I*^*2*^ statistics was used to assess the heterogeneity among the studies. A value of *p* < 0.05, *I*^2^ > 50% of chi-squared test demonstrated obvious heterogeneity among these studies, and a random effect model was applied in this occasion. Otherwise, a model of fixed effect was carried out to assess the pooled results with no obvious heterogeneity. Sensitivity analysis was conducted by omitting one of the included studies respectively to evaluate the certainty of the pooled HRs. Begg’s and Egger’s test was used to estimate the potential publication bias. It is considered statistically significant with *p* < 0.05.

## Results

### Selection and description of enrolled studies

**Fig. 1:**
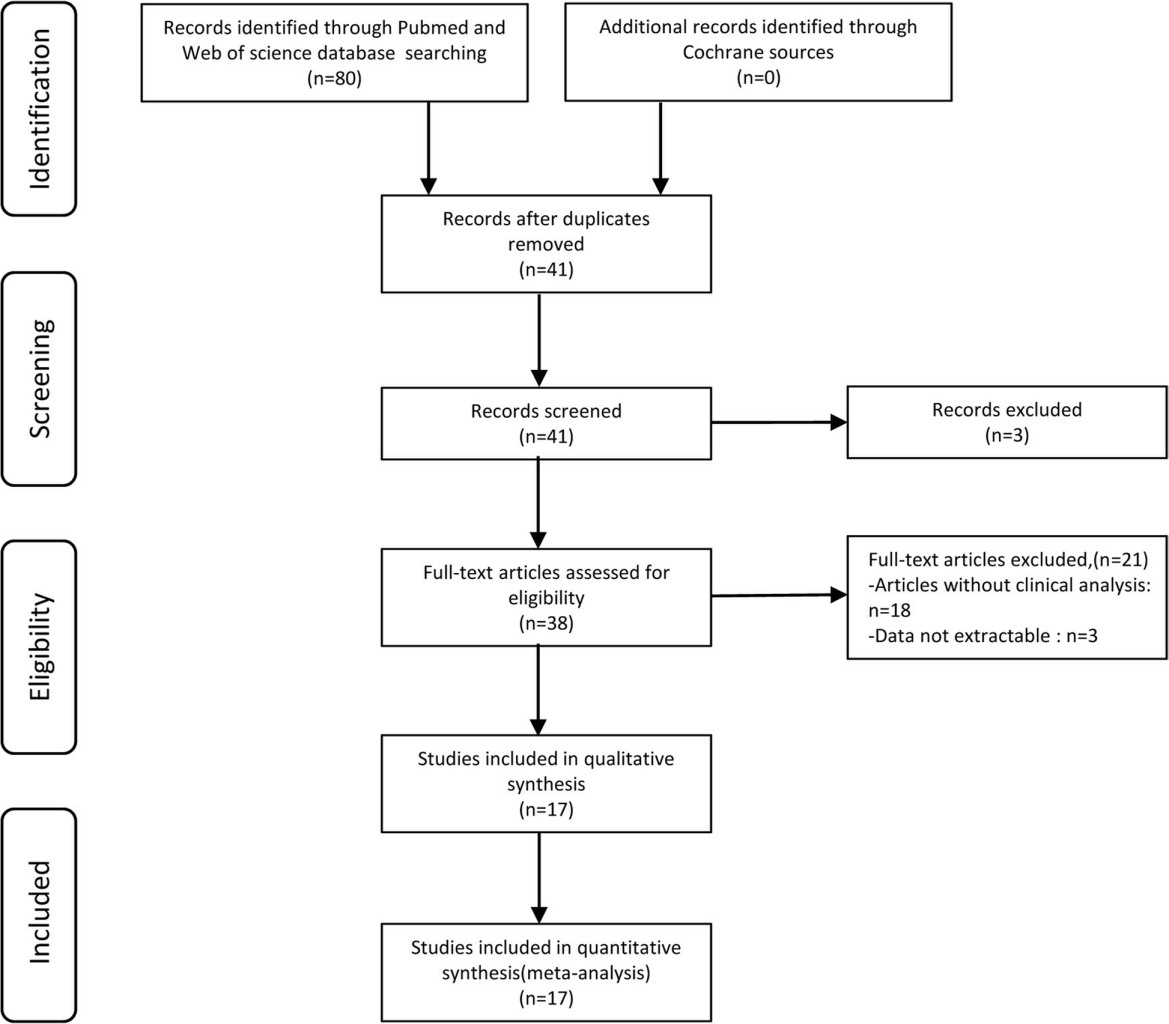
Flow diagram of the study selection procedure

A total of 80 publications were initially searched as potential studies, and 39 duplicates of them were excluded. Then, 3 publications were excluded as review and meta-analysis by screening via the titles and abstracts. Afterwards, 38 full-text articles were thoroughly assessed, of which 18 studies without clinical analyses and 3 studies with unextractable data were removed. As a result, 17 studies comprising 1098 patients were enrolled in this meta-analysis. Of note, all the enrolled patients were from China. The selection process was concisely shown by a flow diagram in Fig. 1.

The detailed data of the enrolled publications were presented in Table 1. All of the articles were performed in China and published between 2017 and 2021. Out of the seventeen studies, seven focused on the HCC. Additionally, there were other seven cancer types including BlC, BrC, CRC, ESCC, GC, LC, and PTC. The circZFR expression level was detected by quantitative real-time polymerase chain reaction (qRT-PCR) with the sample size ranging from 30 to 170, and analyzed via univariate analyses or multivariate analyses. Among the publications, nine studies assessed the relationship between circZFR and clinical stages (TNM stage), eleven studies measured OS, and 10 of whose follow-up time were more than 60 months. Most of the studies adopted the median or mean expression of circZFR as the cut-off value. Patients in 14 studies did not accepted anti-tumor therapy (adjuvant therapy) before the specimen collected. The quality of the enrolled studies was high with all of the NOS scores ≥ 7.

**Table 1:** Summary of the main characteristics of the included studies

Author	Year	Country	Cancer type	Clinical stage	Sample size	Cut-off value	Follow-up (months)	Detection method	Adjuvant therapy	Survival analysis	Outcome measure	NOS
Cedric, B [15]	2020	China	HCC	T1–T4	62	Mean	–	qRT–PCR	None	Univariate	CP	7
Chen, Z [19]	2020	China	BrC	I–IV	70	Median	60	qRT–PCR	N/A	Univariate	OS, CP	9
Fang, N [23]	2020	China	ESCC	I–IV	58	Median	–	qRT–PCR	None	Univariate	CP	7
Huang, S [21]	2020	China	GC	–	60	N/A	60	qRT–PCR	None	Univariate	OS	8
Huang, W [24]	2020	China	BlC	–	55	Median	60	qRT–PCR	None	Univariate	OS, DFS	8
Li, L [25]	2021	China	HCC	I–III	49	N/A	–	qRT–PCR	None	Univariate	CP	7
Lin, Y [26]	2021	China	HCC	I–IV	50	Median	60	qRT–PCR	None	Multivariate	OS,CP	9
Liu, M [20]	2020	China	BrC	I–IV	65	Median	54	qRT–PCR	None	Univariate	OS, CP	8
Liu, W [16]	2018	China	LC	–	44	N/A	80	qRT–PCR	None	Univariate	OS	8
Luo, L [27]	2021	China	BlC	I–IV	60	N/A	78	qRT–PCR	N/A	Univariate	OS,CP	8
Tan, A [28]	2019	China	HCC	–	80	Mean	60	qRT–PCR	None	Univariate	OS	8
Wei, H [17]	2018	China	PTC	–	41	N/A	60	qRT–PCR	N/A	Univariate	OS	8
Xu, R [29]	2021	China	HCC	I–IV	40	N/A	–	qRT–PCR	None	Univariate	CP	7
Yang, X [30]	2019	China	HCC	I–IV	30	Median	–	qRT–PCR	None	Univariate	CP	7
Zhang, P [22]	2017	China	CRC	I–IV	170	N/A	–	qRT–PCR	None	Univariate	CP	7
Zhang, W [18]	2019	China	BlC	Ta–T4	104	Median	72	qRT–PCR	None	Univariate	OS, PFS, CP	9
Zhan, W [31]	2020	China	HCC	I–IV	60	N/A	100	qRT–PCR	None	Univariate	OS, CP	9

*Abbreviations*: *BlC* bladder cancer, *BrC* breast cancer, *CRC* colorectal cancer, *CP* clinicopathological parameters, *DFS* disease-free survival, *ESCC* esophageal squamous cell cancer, *GC* gastric cancer, *HCC* hepatocellular carcinoma, *LC* lung cancer, *N/A* not available, *NOS* Newcastle-Ottawa Scale, *OS* overall survival, *PFS* progression-free survival, *PTC* papillary thyroid cancer, *qRT-PCR* quantitative real-time polymerase chain reaction

### Association between circZFR expression and OS

**Fig. 2:**
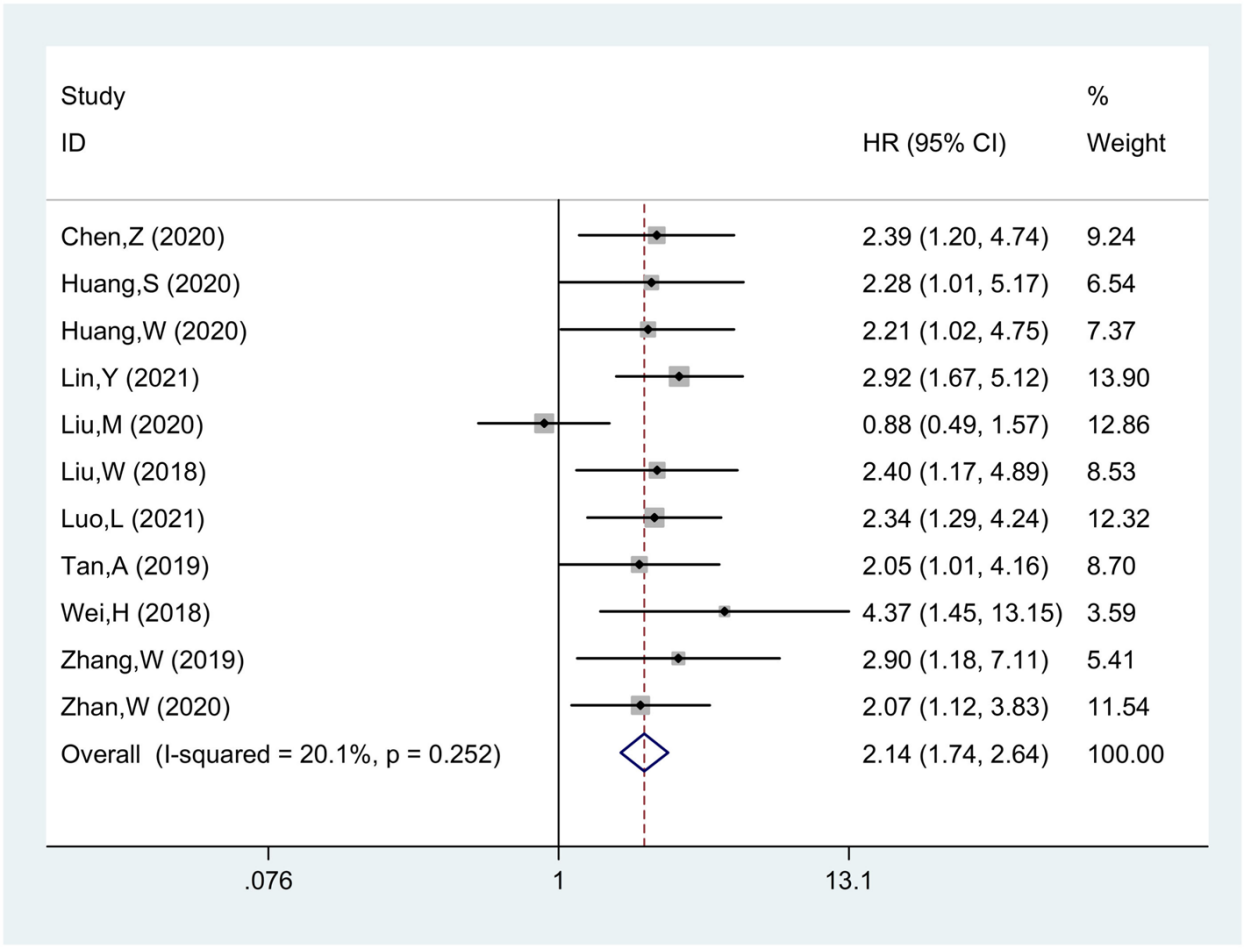
Forest plot evaluating the correlation between circZFR expression and OS

Eleven studies comprising 689 patients were included for pooled OS analysis. With the absence of the heterogeneity (*I*^*2*^ = 20.1%, *p* = 0.252), the fixed effect model was applied to analyze the OS (HR = 2.14, 95% CI 1.74, 2.64), which indicated that high expression of circZFR related to a worse OS (Fig. 2).

Additionally, four stratified analyses were further conducted on sample size (< 60 and ≥ 60), cancer type (BlC, BrC, HCC, and others), follow-up months (< 60 and ≥ 60), and cut-off value (mean, median and not available (N/A)) (Supplementary Figure 1). The detailed pooled HRs and 95% CIs were summarized in Table 2. For the sample size stratified analysis, both the more than 60 (HR = 1.90, 95% CI 1.47, 2.45) and less than 60 (HR = 2.73, 95% CI 1.90, 3.91) groups with circZFR overexpression exhibited an unfavorable OS. Of note, for studies evaluating on cancer type, the results indicated an insignificant relation of the circZFR overexpression to the OS of the BrC (HR = 1.43, 95% CI 0.54, 3.79), while high circZFR level was significantly associated with short survival of BlC (HR = 2.41, 95% CI 1.59, 3.66), HCC (HR = 2.38, 95% CI 1.66, 3.40) and other cancer (HR = 2.65, 95% CI 1.63, 4.29) patients. What is more, the high circZFR expression predicted an unfavorable OS with follow-up time more than 60 months (HR = 2.44, 95% CI 1.95, 3.05), while that less than 60 group showed no significant linkage. According to the cut-off value stratified analysis, whether the cut-off value was mean, median, or N/A, the high circZFR expression indicated worse OS.

**Table 2:** Stratified analysis of the HRs of overall survival

Subgroups	No. of studies	No. of patients	HR (95% CI)	Model	Heterogeneity	
					***I***^**2**^ (%)	***p*** value
1 Sample size						
1.1 ≥ 60	7	499	1.90 (1.47, 2.45)	Fixed	31.6	0.187
1.2< 60	4	190	2.73 (1.90, 3.91)	Fixed	0.00	0.760
2 Cancer type						
2.1 BlC	3	219	2.41 (1.59, 3.66)	Random	0.00	0.895
2.2 BrC	2	135	1.43 (0.54, 3.79)	Random	78.9	0.030
2.3 HCC	3	190	2.38 (1.66, 3.40)	Random	0.00	0.644
2.4 Others	3	145	2.65 (1.63, 4.29)	Random	0.00	0.608
3 Follow-up						
3.1 ≥ 60	10	624	2.44 (1.95, 3.05)	Fixed	0.00	0.987
3.2< 60	1	65	1.38 (0.57, 3.33)	Fixed	–	–
4 Cut-off value						
4.1 Mean	1	80	2.05 (1.01, 4.16)	Fixed	–	–
4.2 Median	5	344	2.38 (1.77, 3.20)	Fixed	0.00	0.826
4.3 N/A	5	265	2.39 (1.73, 3.29)	Fixed	0.00	0.848

*BlC* bladder cancer, *BrC* breast cancer, *CI* confidence interval, *HCC* hepatocellular carcinoma, *HR* hazard ratio, *N/A* not available

### Association between circZFR expression and clinicopathologic parameters

**Fig. 3:**
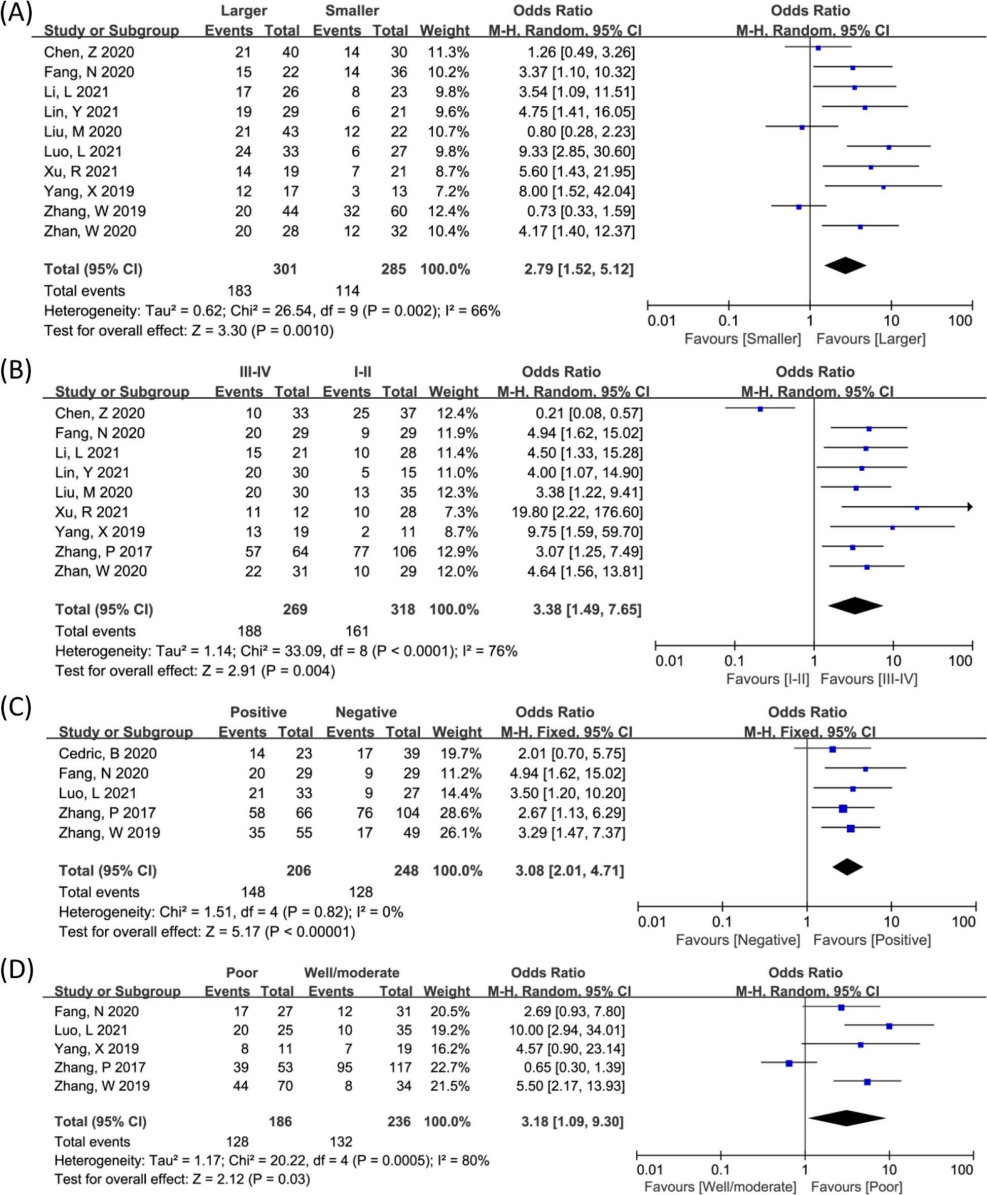
Forest plots of the association between circZFR expression and clinicopathological parameters, including tumor size (**A**), clinical stage (**B**), LNM (**C**), and histology grade (**D**)

Seven items of clinicopathologic parameters including age, gender, tumor size, clinical stage, distant metastasis (DM), lymph node metastasis (LNM), and histology grade were further analyzed to evaluate their correlation with the circZFR expression. Notably, ten studies enrolled to explore the correlation between circZFR expression and tumor size, demonstrating that higher circZFR expression predicted larger tumor size (OR = 2.79, 95% CI 1.52, 5.12) (Fig. 3). Similarly, the upregulation of circZFR expression indicated advanced clinical stage (OR = 3.38, 95% CI 1.49, 7.65), tendency of LNM (OR = 3.08, 95% CI 2.01, 4.71) and higher histology grade (OR = 3.18, 95% CI 1.09, 9.30). As shown in Supplementary Figure 2, insignificant association found between circZFR expression and age (OR = 1.16, 95% CI 0.86, 1.57), gender (OR = 1.02, 95% CI 0.73, 1.43), and DM (OR = 1.39, 95% CI 0.49, 3.94). The detailed data were summarized in Table 3.

**Table 3:** Association between circZFR and other clinicopathologic parameters

Outcome or subgroup	Studies	Participants	Odds ratio (95% CI)	***P*** value	Model	Heterogeneity	
						***P*** value	***I***^**2**^
Age	11	778	1.16 (0.86, 1.57)	0.33	Fixed	0.20	25%
Gender	10	683	1.02 (0.73, 1.43)	0.89	Fixed	0.60	0%
Tumor size	10	586	2.79 (1.52, 5.12)	0.001	Random	0.002	66%
Clinical stage	9	587	3.38 (1.49, 7.65)	0.004	Random	< 0.0001	76%
DM	4	367	1.39 (0.49, 3.94)	0.53	Random	0.007	75%
LNM	5	454	3.08 (2.01, 4.71)	< 0.00001	Fixed	0.82	0%
Histology grade	5	422	3.18 (1.09, 9.30)	0.03	Random	0.0005	80%

*CI* confidence interval, *DM* distant metastasis, *LNM* lymph node metastasis, *OR* odds ratio

### Sensitivity analysis and publication bias

We performed the sensitivity analysis by calculating the HRs and 95% CIs after excluding each of the enrolled study, and the result indicated the stability of the pooled HRs and corresponding 95% CIs of the association between circZFR expression and OS (Supplementary Figure 3A).

The potential publication bias was measured by Begg’s and Egger’s test. And there was no significant publication bias according to the symmetrically distributed funnel plot (Supplementary Figure 3B). Furthermore, the Begg’s and Egger’s test (*p* = 0.276 and *p* = 0.173) demonstrated the absence of the publication bias.

### The regulation mechanism of circZFR in cancers

**Fig. 4:**
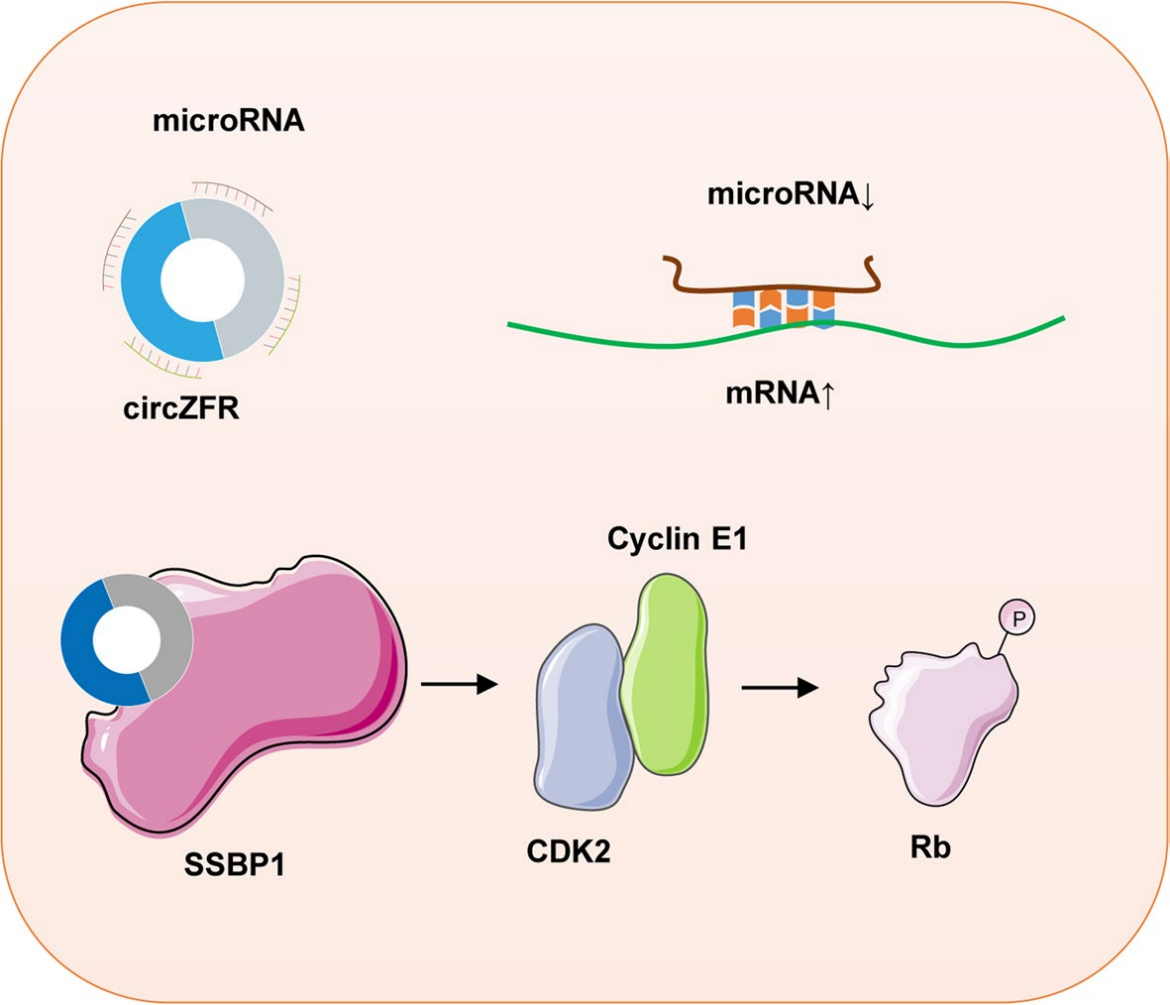
The major mechanism patterns of circZFR in regulating cancers development

Recently, multiple studies have reported the potential mechanism of circZFR for proliferation, apoptosis, migration, invasion, and metabolism in various cancers. Currently, ceRNA mechanism has been focused on and partially illuminated in regulating cancers development. For example, the circZFR/miR-545-3p/CBLL1 axis, circZFR/miR-195-5p/KPNA4 axis, and circZFR/miR-4302/ZNF121axis promoted the growth, migration, invasion, EMT, and chem-resistance, arrested cell cycle and inhibited apoptosis in LC [16, 32, 33]. Specially, HCC tumor cell-derived exosome suppressed the metastasis of HCC mediating the degradation of miR-375 via circZFR and upregulated MMP-16 [26]. Moreover, circZFR can bind to the cellular protein directly and interfere its function. It has been reported that circZFR decreased H3K4me3 levels on the CCND1 promoter to promote the tumorigenic capacity in LC [34]. Besides, circZFR bound with SSBP1, thereby inducing the assembly of CDK2/cyclin E1 complexes, which induced p-Rb phosphorylation, thus releasing activated E2F1 leading to cell cycle progression and cell proliferation in cervical cancer [35]. We have reviewed the reported mechanism about circZFR in Table 4 and Fig. 4.

**Table 4:** The ceRNA regulation of circZFR in various cancers

Cancer type	miRNA	mRNA	Function
BlC	miR-516a-5p	FBXL18	Growth, metastasis, chemo-resistance [24]
	miR-377	ZEB2	Cell growth, migration, invasion, cell cycle, apoptosis [18]
BrC	miR-578	HIF1A	Proliferation, apoptosis, migration, invasion, ATP levels [19]
	miR-532-3p	–	Proliferation, migration, invasion, EMT [20]^a^
	–	PI3K/AKT	Proliferation, apoptosis [36]
CRC	miR-532-3p	FOXO4	Proliferation, migration [37]
ESCC	miR-377	VEGF	Proliferation, migration, invasion [23]
GC	miR-101-3p	–	Migration, invasion [21]
	miR-130a，miR-107	PTEN	Proliferation, cell cycle, apoptosis [38]^a^
HCC	miR-1270	PLAGL2	Proliferation, migration, invasion, EMT [39]
	–	MAP2K1	Proliferation, stemness [15]
	miR-620	–	Proliferation, migration, invasion [40]^a^
	miR-3619-5p	CTNNB1, Wnt/β-catenin pathway	Proliferation, EMT [28]
	miR-375	HMGA2	Proliferation, glycolytic metabolism, apoptosis [29]
	miR-511	AKT1	Proliferation, migration, invasion, apoptosis [30]
	miR-377-3p	FGFR1	Proliferation, cycle progression, migration [31]
LC	miR-4302	ZNF121-dependent MYC expression	Proliferation, invasion [16]
	miR-377-3p	GOT1	Cisplatin-resistance, proliferation, viability, apoptosi s[41]
	miR-377-5p	NOVA2	Proliferation, motility [32]
	miR-545-3p	CBLL1	Growth, apoptosis, cell cycle, migration, invasion [42]
	miR-195-5p	KPNA4	Paclitaxel resistance, cell cycle, apoptosis [14]
	miR-101-3p	CUL4B	Proliferation, migration, invasion [33]
PTC	miR-1261	C8orf4	Proliferation, migration, invasion [17]
RCC	miR-206	Met-Wnt/β-catenin and PI3K/AKT	Growth, migration, invasion [43]

*Abbreviations*: *BlC* bladder cancer, *BrC* breast cancer, *CRC* colorectal cancer, *ESCC* esophageal squamous cell cancer, *GC* gastric cancer, *HCC* hepatocellular carcinoma, *LC* lung cancer, *PTC* papillary thyroid cancer, *RCC* renal cell carcinoma

^a^circZFR acts as tumor suppressor genes

## Discussion

CircRNAs have been reported as the clinical diagnostic biomarkers and treatment targets in terms of stability, specificity and conservation [44]. Isolation and detection of circRNAs from peripheral blood or tissue is feasible. A pan-cancer analysis of RNA sequencing of thousands of human cancer genomes verified that ZFR is a potential potent cancer driver gene. CircZFR is a circular RNA derived from ZFR exons via back-splicing. According to the majority of studies, circZFR was overexpressed in various cancer, including BlC, BrC, HCC, LC, and renal cell carcinoma [15–17, 21, 28]. But a few studies identified that circZFR was downregulated in cancer tissues like BrC and colorectal cancer compared with adjacent tissues [20, 22, 37]. It suggested the expression heterogeneity of circZFR in different tumor tissues. CircZFR has been proved the significant association with clinical characteristics and prognostic parameters in various cancer.

Here, we first estimated correlation of circZFR expression with prognosis of cancer patients. A total of 689 patients from eleven studies were included for pooled OS analysis. The pooled HR demonstrated that high circZFR expression indicated a poor OS without significant heterogeneity. In fact, the cut-off value of the included studies were inconsistent. Some cut-off values were median, some were mean, and the rest studies that did not report cut-off values were labeled ‘NA’. As the different cut-off values may affect the result, we made a stratified analysis. And according to the results, all of the three groups (mean, median, and N/A) with high circZFR expression exhibited poor OS. That means, the inconsistency of the cut-off value in this study wouldn’t affect the results of OS analysis. Then we evaluated the association between circZFR and the major clinical characteristics including age, gender, tumor size, clinical stage, DM, LNM, and histology grade. The higher circZFR expression predicted larger tumor size, advanced clinical stage, higher possibility of LNM, and higher histology grade. No significant correlation between circZFR expression and age, gender, and DM. More studies need to be included to analyze the heterogeneity of pooled HR for tumor size, clinical stage, and DM.

At present, circRNAs play their biological functions mainly through the following four patterns, including acting as miRNA molecular sponge through the mechanism of ceRNAs, interacting with RNA-binding proteins (RBPs), regulating of splicing or transcription of genes, translation into proteins or small peptides. However, the function and mechanism of circZFR in the development and progression of cancers still have not been clear deeply. We found the major mechanism of circZFR regulating development and progression in cancers was ceRNA [17, 43]. Only one study showed the function of circZFR in hepatic carcinoma cells-derived exosome suppressing migration and invasion [26]. Only a few studies reported circZFR promoted cancers via interacting with RBPs [35] or regulating the chromatin modification at the transcription start site of genes [34]. More studies are needed to put into circZFR regulation mechanism.

However, this study also has several limitations. Firstly, almost all included patients were from China, so this reduced the applicability of the results across different races and regions. Then, we only enrolled 11 studies in the prognosis meta-analysis, which limited the wide application of the meta-analysis results. Moreover, more studies needed to be included to analyze the correlation between circZFR expression and age, gender, and DM. Finally, because several studies did not report HRs with 95% CIs in the article, we had to extract and calculate them on the basis of the Kaplan-Meier curves.

## Conclusions

Taken together, this study demonstrated that high circZFR expression was remarkably correlated with unfavorable OS, as well as clinicopathological parameters including larger tumor size, advanced clinical stage, higher possibility of LNM, and higher histology grade in Chinese cancer patients. Therefore, circZFR can be served as a prognostic biomarker of cancer. Nevertheless, further large-scale studies from different ethnic population are needed to verify the results.

## Supplementary Information

**Additional file 1: Supplementary Figure 1**. Forest plots of the subgroups analysis evaluating the correlation between circZFR expression and OS, including sample size (A), cancer type (B), follow-up months (C), and cut-off value (D). **Supplementary Figure 2**. Forest plots evaluating the correlation between circZFR expression and other clinicopathological parameters, including age (A), gender (B) and DM (C). **Supplementary Figure 3**. Sensitivity analysis (A) and funnel plot for publication bias (B) for circZFR on OS.

**Additional file 2: Supplementary table**. Results of quality assessment using Newcastle-Ottawa Scale (NOS) score for the enrolled studies.

**Additional file 3.** Search strategy.

## Data Availability

The dataset used and analyzed during the current study are available from the corresponding author on reasonable request.

## Abbreviations

BlC: Bladder cancer
BrC: Breast cancer
circZFR: Circular RNA zinc finger RNA-binding protein
CI: Confidence interval
circRNAs: Circular RNAs
CeRNA: Competing endogenous RNA
CRC: Colerectal cancer
CP: Clinicopathological parameters
DFS: Disease-free survival
DM: Distant metastasis
DZF: Domain associated with zinc fingers
ESCC: Esophageal squamous cell cancer
GC: Gastric cancer
HCC: Hepatocellular carcinoma
HR: Hazard ratio
LC: Lung cancer
LNM: Lymph node metastasis
N/A: Not available
ncRNA: Non-coding RNA
NOS: Newcastle-Ottawa Scale
OR: Odds ratio
OS: Overall survival
PFS: Progression-free survival
PTC: Papillary thyroid cancer
qRT-PCR: Quantitative real-time polymerase chain reaction
RBPs: RNA-binding proteins
