# Supplementary material for: Prognostic and clinicopathologic significance of circZFR in multiple human cancers
Source: World J Surg Oncol. 2022 Aug 26;20:268. doi: 10.1186/s12957-022-02733-9 (PMC9413939; doi:10.1186/s12957-022-02733-9)
Supplement: Supplementary file 1 — Additional file 1: Supplementary Figure 1. Forest plots of the subgroups analysis evaluating the correlation between circZFR expression and OS, including sample size (A), cancer type (B), follow-up months (C), and cut-off value (D). Supplementary Figure 2. Forest plots evaluating the correlation between circZFR expression and other clinicopathological parameters, including age (A), gender (B) and DM (C). Supplementary Figure 3. Sensitivity analysis (A) and funnel plot for publication bias (B) for circZFR on OS. [file 12957_2022_2733_MOESM1_ESM.docx]

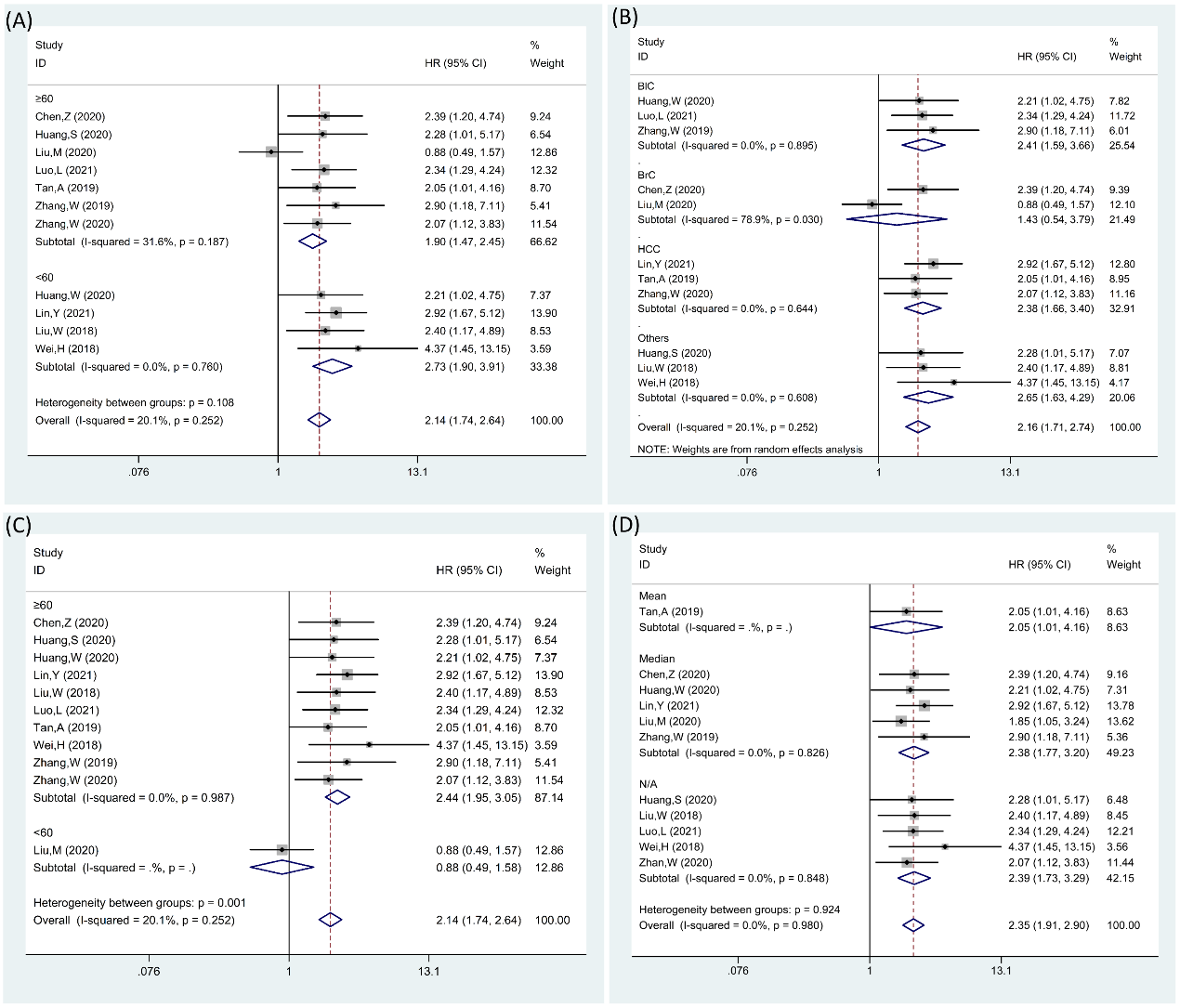


**Supplementary figure 1.** Forest plots of the subgroups analysis evaluating the correlation between circZFR expression and OS, including sample size (A), cancer type (B), follow up months (C), and cut-off value (D)..


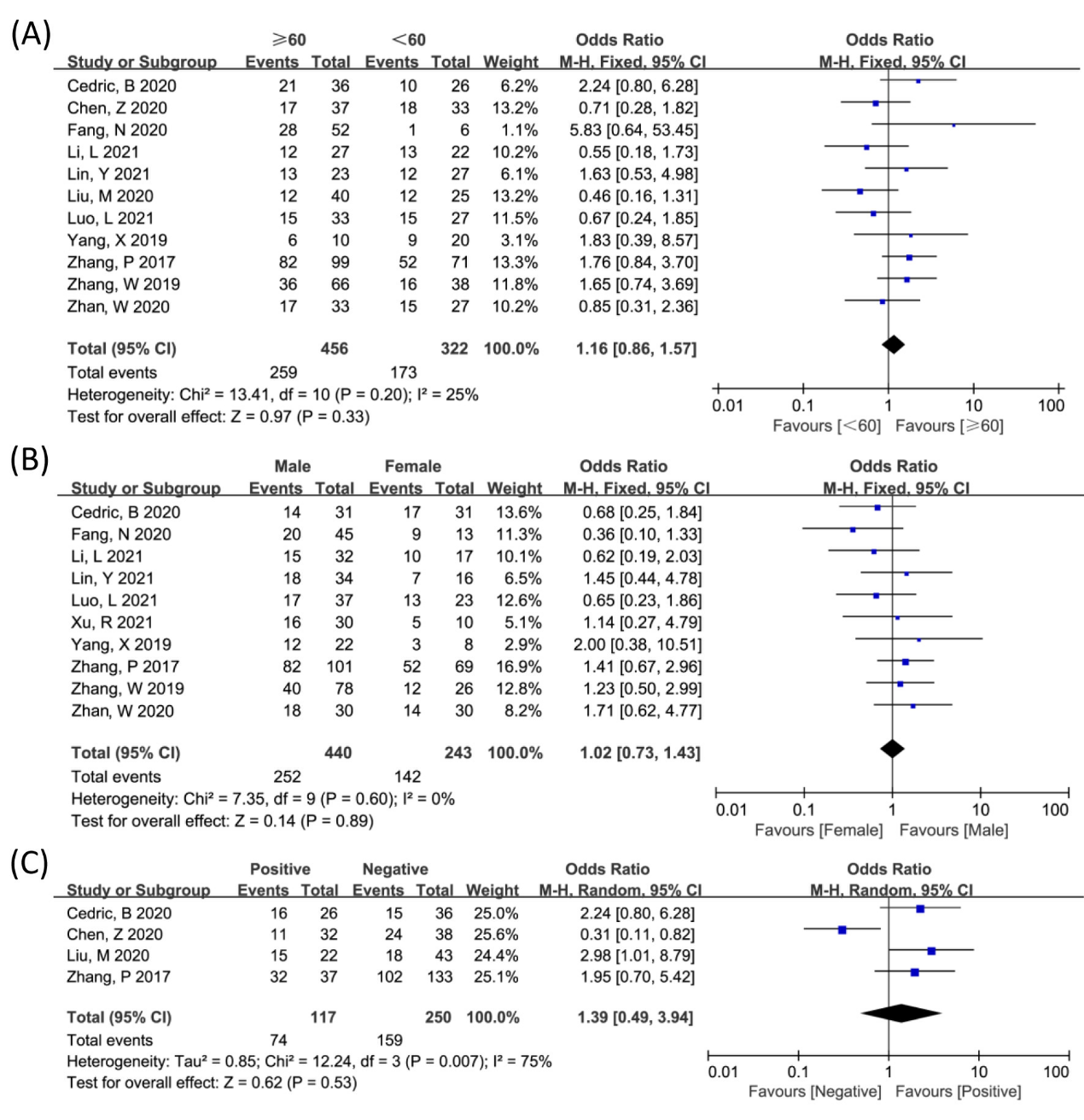


**Supplementary figure 2.** Forest plots evaluating the correlation between circZFR expression and other clinicopathological parameters, including age (A), gender (B) and DM (C).


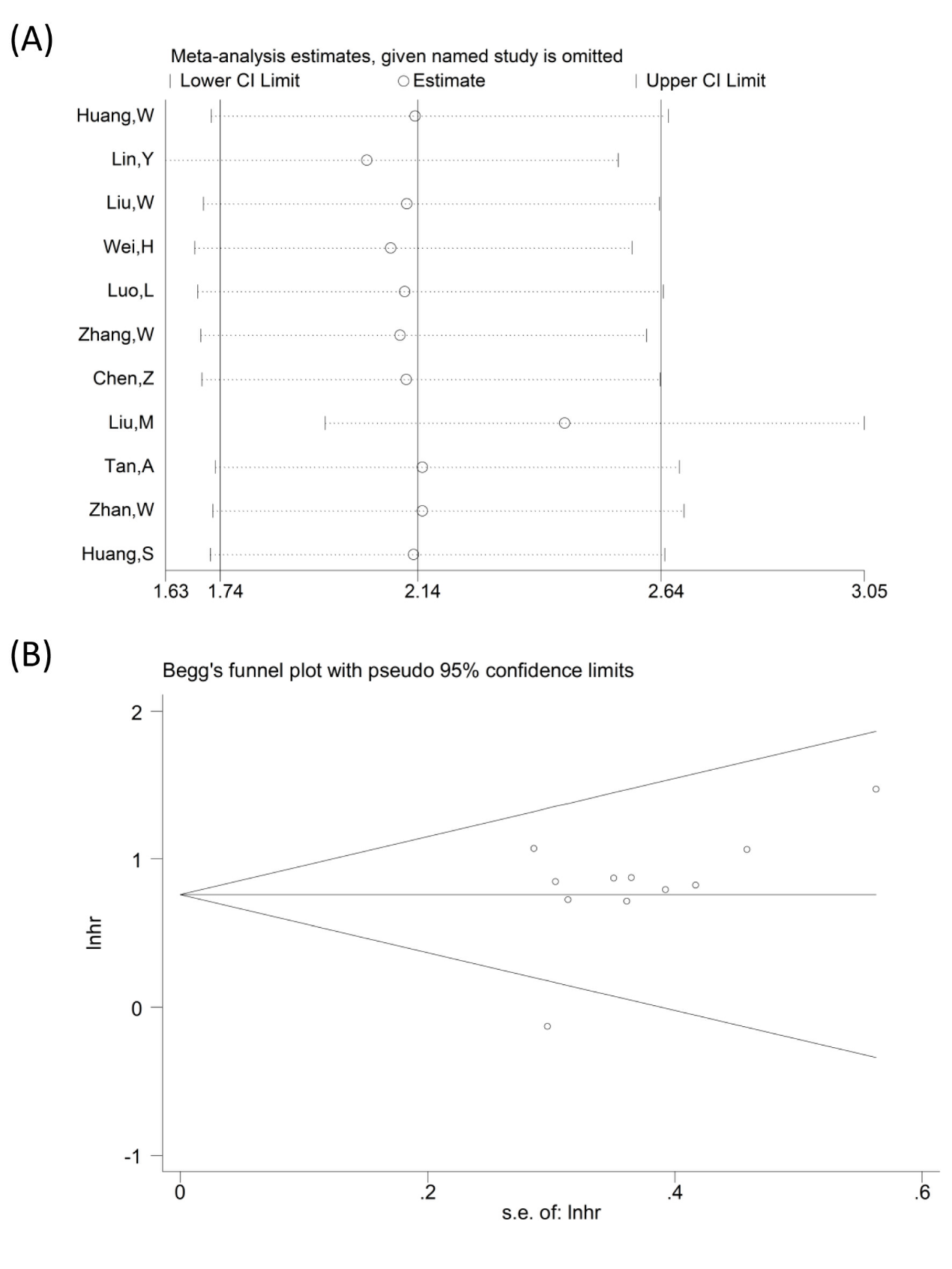


**Supplementary figure 3.** Sensitivity analysis (A) and funnel plot for publication bias (B) for circZFR on OS.
