# Supplementary material for: Prognostic and clinicopathologic significance of circZFR in multiple human cancers
Source: World J Surg Oncol. 2022 Aug 26;20:268. doi: 10.1186/s12957-022-02733-9 (PMC9413939; doi:10.1186/s12957-022-02733-9)
Supplement: Supplementary file 2 — Additional file 2: Supplementary table. Results of quality assessment using Newcastle-Ottawa Scale (NOS) score for the enrolled studies. [file 12957_2022_2733_MOESM2_ESM.docx]

**Supplementary table. Results of quality assessment using Newcastle-Ottawa Scale (NOS) score for the enrolled studies**

| First author | Selection | | | |  | Comparability |  | Outcome | | | NOS score |
| --- | --- | --- | --- | --- | --- | --- | --- | --- | --- | --- | --- |
|  | Representativeness of the exposed cohort | Selection of the non exposed cohort | Ascertainment of exposure | Demonstration that outcome of interest was not present at start of study |  | Comparability of cohorts on the basis of the design or analysis |  | Assessment of outcome | Was follow-up long enough for outcomes to occur | Adequacy of follow up of cohorts |  |
| Cedric, B | ★ | ★ | ★ | ★ |  | ★★ |  | ★ |  |  | 7 |
| Chen, Z | ★ | ★ | ★ | ★ |  | ★★ |  | ★ | ★ | ★ | 9 |
| Fang, N | ★ | ★ | ★ | ★ |  | ★★ |  | ★ |  |  | 7 |
| Huang, S | ★ | ★ | ★ | ★ |  | ★ |  | ★ | ★ | ★ | 8 |
| Huang, W | ★ | ★ | ★ | ★ |  | ★ |  | ★ | ★ | ★ | 8 |
| Li, L | ★ | ★ | ★ | ★ |  | ★★ |  | ★ |  |  | 7 |
| Lin, Y | ★ | ★ | ★ | ★ |  | ★★ |  | ★ | ★ | ★ | 9 |
| Liu, M | ★ | ★ | ★ | ★ |  | ★★ |  | ★ |  | ★ | 8 |
| Liu, W | ★ | ★ | ★ | ★ |  | ★ |  | ★ | ★ | ★ | 8 |
| Luo, L | ★ | ★ | ★ | ★ |  | ★ |  | ★ | ★ | ★ | 8 |
| Tan, A | ★ | ★ | ★ | ★ |  | ★ |  | ★ | ★ | ★ | 8 |
| Wei, H | ★ | ★ | ★ | ★ |  | ★ |  | ★ | ★ | ★ | 8 |
| Xu, R | ★ | ★ | ★ | ★ |  | ★★ |  | ★ |  |  | 7 |
| Yang, X | ★ | ★ | ★ | ★ |  | ★★ |  | ★ |  |  | 7 |
| Zhan, W | ★ | ★ | ★ | ★ |  | ★★ |  | ★ | ★ | ★ | 9 |
| Zhang, P | ★ | ★ | ★ | ★ |  | ★★ |  | ★ |  |  | 7 |
| Zhan, W | ★ | ★ | ★ | ★ |  | ★★ |  | ★ | ★ | ★ | 9 |
