# Supplementary material for: Prognostic and clinicopathologic significance of circZFR in multiple human cancers
Source: World J Surg Oncol. 2022 Aug 26;20:268. doi: 10.1186/s12957-022-02733-9 (PMC9413939; doi:10.1186/s12957-022-02733-9)
Supplement: Supplementary file 3 — Additional file 3. Search strategy. [file 12957_2022_2733_MOESM3_ESM.docx]

**Search strategy of Pubmed:** ((((((((((circZFR) OR (circ_ZFR)) OR (circ-ZFR)) OR (circRNA ZFR)) OR (circular RNA ZFR)) OR (circ_0072088)) OR (circRNA_103809)) OR (hsa_circRNA_103809)) OR (circular RNA hsa_circRNA_103809)) OR (circ_103809)) OR (circ_0072083).

**Search strategy of Web of Science:** ((((((((((TS=( circZFR)) OR TS=( circ_ZFR)) OR TS=( circ-ZFR)) OR TS=((circRNA ZFR)) OR TS=(circular RNA ZFR)) OR TS=(circ_0072088)) OR TS=(circRNA_103809)) OR TS=(hsa_circRNA_103809)) OR TS=(circular RNA hsa_circRNA_103809)) OR TS=(circ_103809)) OR TS=(circ_0072083).

**Search strategy of The Cochrane Library:** ((circZFR) OR (circ_ZFR) OR (circ-ZFR) OR (circRNA ZFR) OR (circular RNA ZFR) OR (circ_0072088) OR (circRNA_103809) OR (hsa_circRNA_103809) OR (circular RNA hsa_circRNA_103809) OR (circ_103809) OR (circ_0072083)):ti,ab,kw.
